# Strictinin: A Key Ingredient of Tea

**DOI:** 10.3390/molecules28093961

**Published:** 2023-05-08

**Authors:** Jason T. C. Tzen

**Affiliations:** Graduate Institute of Biotechnology, National Chung-Hsing University, Taichung 402, Taiwan; tctzen@dragon.nchu.edu.tw; Tel.: +886-4-22840328 (ext. 776); Fax: +886-4-22853527

**Keywords:** ellagitannin, functional activities, practical applications, Pu’er tea, strictinin

## Abstract

Strictinin is a relatively tiny ellagitannin, which is found in many plants as a minor constituent. Catechins are known as the major constituents in the young leaves of most tea plants, while strictinin was found as a major constituent in the Pu’er tea plant. In some Pu’er tea varieties, strictinin was identified as the most abundant phenolic compound rather than catechins. In the past decade, strictinin was demonstrated to possess several functional activities, including antiviral, antibacterial, anti-obesity, laxative, anticaries, anti-allergic, antipsoriatic, antihyperuricemia, antidiabetic, and anticancer effects. These functional activities were in accordance with the therapeutic effects empirically perceived for Pu’er tea. Evidently, strictinin is the key ingredient in Pu’er tea that acts as a herbal medicine. In functionally-based applications, an instant powder of Pu’er tea infusion was formulated as an active raw material to be supplemented in food, cosmetics, and beverages; a new type of tea named Bitter Citrus Tzen Tea was developed by combining three teas empirically consumed to expel the cold, and new edible oral care products were designed for caries prevention by supplementation with Pu’er tea extract. More functional activities and practical applications of strictinin are scientifically anticipated in follow-up research.

## 1. Introduction

Strictinin, a hydrolysable tannin belonging to the family of ellagitannin, was first identified in the leaves of *Casuarina stricta* and *Stachyurus praecoxwith* by Okuda’s group in 1982 [1,2], with an IUPAC name of β-D-Glucopyranose 4,6-(4,4′,5,5′,6,6′-hexahydroxy [1,1′-biphenyl]-2,2′-dicarboxylate) 1-(3,4,5-trihydroxybenzoate). As described in the work, seven ellagitannins were identified in two species with five novel compounds, casuarinin, casauriin, stachyurin, casuarictin, and strictinin, apparently termed in relation to their species names. Thereafter, strictinin was occasionally detected as a minor active constituent in several plant species, such as *Callistemon subulatus*, strawberries (*Fragaria* × *ananassa* Duch.), *Platycarya strobilacea*, etc. [3,4,5]. Moreover, instead of isolation from natural sources, high-yield production of strictinin by chemical synthesis was also successfully achieved via intramolecular coupling of gallates [6]. 

Tea is a significant dietary source of ellagitannins [7]. Similar to many other plants, strictinin was found to be a minor constituent in most tea plant species, in which catechins represented the major constituents [8,9]. Unexpectedly, strictinin was identified as a major phenolic compound in Pu’er tea, and the content of strictinin varied significantly, as detected in tea samples collected from different areas of Yunnan, China [10]. Naturally, wild Pu’er tea plants grow as huge trees. A large number of wild Pu’er tea trees are found in the uninhabited forests of Yunnan, and some wild Pu’er trees are also sporadically scattered in the whole province as well as in the mountain areas of several neighboring countries, such as Burma, Laos, Vietnam, and Thailand. To massively produce Pu’er tea for commercial benefit, Pu’er tea plants are condensed to shrubs and densely cultivated in the terraced farms of Yunnan. The annual production of Pu’er tea was higher than 150,000 tons and is predicted to be steadily increased over the next couple of decades (www.huaon.com, accessed on 25 March 2023). In general, the content of strictinin in Pu’er tea prepared from wild trees is higher than from cultivated shrubs.

**Figure 1:**
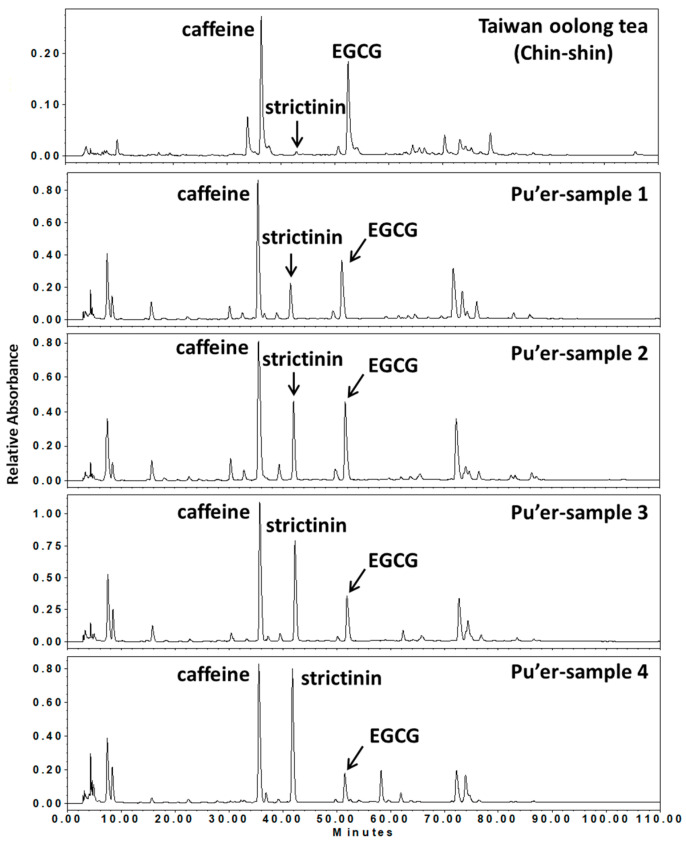
Chromatograms of infusions from Taiwan oolong tea (Chin-shin) and four Pu’er tea samples from Yunnan, China, by high-performance liquid chromatography (HPLC). Chemical constituents in the infusions of teas were analyzed and compared by HPLC (0–120 min). The peaks of caffeine, strictinin, and (−)-epigallocatechin gallate (EGCG) in the HPLC profiles were indicated. (Adopted and modified from Figure 2 of Chen et al., *J. Food Drug Anal.* **2015**, *23*, 116–123 [10] and Figure 3 of Chen et al., *J. Agric. For.* **2014**, *63*, 129–137 [11]).

Comparably, the level of strictinin in the popular Taiwan oolong tea was nearly undetectable, while the levels in Pu’er tea could be as high as 10% by dry weight, representing the most abundant phenolic compound instead of catechins (Figure 1). Obviously, Pu’er tea is an adequate raw material for the isolation of strictinin from natural sources. Over the past few years, our studies have focused on the functional activities of strictinin, based on the therapeutic effects empirically perceived by the consumption of Pu’er tea. Consequently, strictinin was demonstrated to possess antiviral, antibacterial, anti-obesity, laxative, anticaries, antipsoriatic, and antihyperuricemia effects. In this review, these functional activities of strictinin together with its three other activities, anti-allergic, antidiabetic, and anticancer effects, recently proposed by other groups, are summarized.

## 2. Functional Activities of Strictinin

### 2.1. Antiviral Activity

Antiviral activity of strictinin isolated from green tea was first demonstrated by Suzuki’s group in 2010, who showed that the replication of human, duck, and swine influenza A viruses as well as influenza B virus and human parainfluenza virus (type 1) could be inhibited by strictinin at non-toxic concentrations in vitro [12]. According to their results, the authors proposed that strictinin might directly react with the viral particles to prevent viral invasion in the initial stage. To further detect the putative target of strictinin on the viral invasion at the initial stage, receptor binding and sialidase activity assays of influenza A viruses were examined in the presence of strictinin. Unfortunately, no detectable interference was observed for strictinin on the viral binding and sialidase activity. Therefore, it was postulated that strictinin might exhibit antiviral activity by binding to the virus and/or the surface proteins of the host cell membrane to prevent the entrance of the virus in a manner similar to other polyphenols previously proposed by Haslam [13].

**Figure 2:**
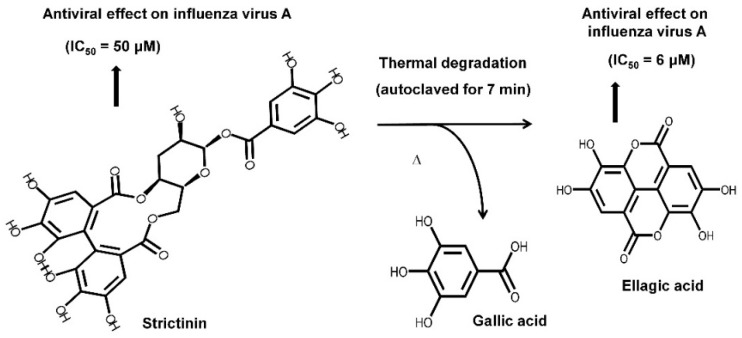
Chemical structures of strictinin and its thermally degraded products, gallic acid, and ellagic acid. (Adopted and modified from Figure 4 of Chen et al., *J. Food Drug Anal.* **2015**, *23*, 116–123 [10]).

Instead of being a minor constituent in green tea, strictinin was found as a major phenolic compound in Pu’er tea; expectably, strictinin isolated from Pu’er tea was shown to possess inhibitory activities on human influenza virus A/Puerto Rico/8/34 [10]. Moreover, strictinin was shown to be thermally labile and apparently degraded after heating at temperatures higher than 80 °C. Strictinin, either in an isolated form or accompanied with other phenolic compounds in tea infusion, was completely decomposed to ellagic acid and gallic acid when it was autoclaved at 121 °C for 7 min (Figure 2). Surprisingly, ellagic acid was found to possess higher inhibitory potency against the human influenza virus A/Puerto Rico/8/34 than strictinin. According to this observation, it was suggested that heating decomposition of strictinin into ellagic acid and gallic acid during the manufactory production of Pu’er tea might be advantageous in terms of enhancing antiviral activity.

According to the experience of Aboriginal people in certain uncultivated mountain areas of Yunnan, China, Pu’er Kucha tea was assumed to be better than Pu’er tea in terms of its therapeutic effects on the common cold [14]. On the basis of morphological similarity, the tea plant (*Camellia assamica* var. kucha Chang et Wang) used for the preparation of Pu’er Kucha tea was speculated as a mutant variety of the wild Pu’er tea plant (*Camellia sinensis* var. *assamica*) [15]. This speculation was further supported by the chemical analysis showing that similar profiles of phenolic constituents, including a high abundance of strictinin, were detected in the infusions of Pu’er Kucha tea and Pu’er tea [16]. Nevertheless, two extra compounds, theacrine and chlorogenic acid, with relatively high abundance were found only in Pu’er Kucha tea. Both theacrine and strictinin were shown to possess an inhibitory potency against the human influenza virus A/Puerto Rico/8/34, while chlorogenic acid only displayed weak inhibition. It was concluded that theacrine and strictinin were active ingredients in the anti-influenza activity of Pu’er Kucha tea. Relatively, strictinin possessed higher inhibitory potency against the human influenza viruses A/Puerto Rico/8/34 than theacrine. However, it was possible that theacrine might additionally enhance the anti-influenza activity of Pu’er Kucha tea via activation of physiological or immunological regulation [17]. Obviously, additive or synergistic effects on the antiviral activity of strictinin are expected when it is utilized in combination with some other functional ingredients.

In the history of humans, viral diseases frequently spread rapidly and occasionally caused disastrous damage to human society. Around two decades ago, severe acute respiratory syndrome (SARS) caused by the SARS coronavirus (SARS-CoV) quickly spread all over the world with a mortality rate of 10–15% [18]. In the past few years, the whole world has been continually threatened by the COVID-19 outbreak, caused by a new type of β-coronavirus, named SARS-CoV-2 [19]. Although specific vaccines were developed for SARS-CoV-2, mutant viruses were observed after vaccination, with some of them subsequently becoming the prevailing strains. Searching for natural ingredients with a broad spectrum of antiviral activities might be an adequate solution for COVID-19 treatment. On the basis of their anti-influenza activities reported in the studies of Pu’er tea, theacrine, strictinin, and ellagic acid (thermally degraded product of strictinin) were subjected to the examination of their anti-coronavirus activities by using mouse hepatitis virus, a β-coronavirus classified in the same subgenus as SARS-CoV and SARS-CoV-2 [20]. The inhibitory potency was monitored in three assays, plaque formation ability, viral protein production, and viral progeny. Unexpectedly, strictinin, yet not theacrine and ellagic acid, possessed effective inhibition of mouse hepatitis virus on the infection of Mouse L Cells. In a more detailed analysis, significant inhibition on the infection by the mouse hepatitis virus was observed in the co-treatment or post-treatment of strictinin, while no significant effect on the infection was observed following the pretreatment of strictinin. It seemed that strictinin possessed a broad spectrum of antiviral activities. In a theoretical study to identify candidate compounds against three targets (spike protein, nucleocapsid protein, and 2′-*O*-ribose methyltransferase) of the SARS-CoV-2 coronavirus by combined virtual screening and supervised machine learning, strictinin was simulated to bind to the spike protein and nucleocapsid [21]. It remains to be verified by experimental evidence that the interaction between strictinin and the two target proteins of SARS-CoV-2 coronavirus occurs.

**Figure 3:**
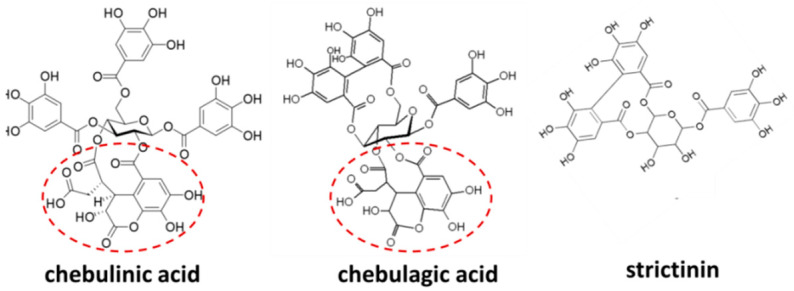
Chemical structures of chebulinic acid, chebulagic acid, and strictinin. The structures of chebulinic acid and chebulagic acid were downloaded from Wikipedia (https://en.wikipedia.org/wiki/Chebulinic_acid#/media/File:Chebulinic_acid.svg, accessed on 25 March 2023) and Wikimedia (https://commons.wikimedia.org/wiki/File:Chebulagic_acid.PNG, accessed on 25 March 2023). The structure of strictinin was downloaded from ResearchGate (https://www.researchgate.net/figure/e-Chemical-structure-of-strictinin_fig1_315370475/download, accessed on 25 March 2023). The chebuloyl moiety found in chebulinic acid and chebulagic acid, yet not in strictinin, is circled by red-dashed lines.

Similarly, chebulinic acid, chebulagic acid, and punicalagin, three ellagitannins with chemical structural analogs to strictinin, also displayed a broad spectrum of antiviral activities by non-specifically interacting with surface glycoprotein–glycosaminoglycan in host cells or allosterically inhibiting 3C-like proteases [22,23,24,25]. However, recent experimental data showed that chebulinic acid and chebulagic acid did not impede the entry or RNA replication of influenza A virus, yet acted as neuraminidase inhibitors to block the virus release [26]. A common chebuloyl moiety in both chebulinic acid and chebulagic acid was proposed to be the key structure in the inhibition of neuraminidase. However, the chebuloyl moiety is not present in strictinin, a relatively tiny and simple ellagitannin (Figure 3). Presumably, the broad-spectrum antiviral activity exhibited by strictinin was not executed via inhibiting neuraminidase to prevent virus budding from the host cells.

### 2.2. Antibacterial Activity

**Figure 4:**
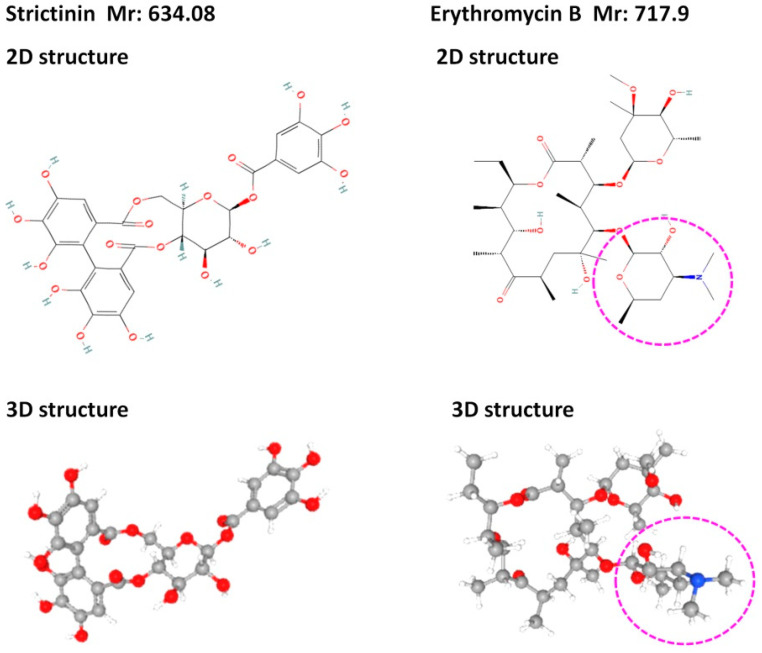
Comparison of structures of strictinin and erythromycin B. The structures were downloaded from the PubChem database from the National Center for Biotechnology Information in the National Library of Medicine: 2D and 3D structures of strictinin from https://pubchem.ncbi.nlm.nih.gov/compound/Strictinin, accessed on 25 March 2023, and those of erythromycin B from https://pubchem.ncbi.nlm.nih.gov/compound/9918244, accessed on 25 March 2023. Relatively, an extra portion with a nitrogen atom (blue color) present in erythromycin B was circled by pink-dashed lines.

The first idea to examine the antibacterial activity of strictinin originated from its structural resemblance to erythromycin (Figure 4), a type of macrolide antibiotic commonly used to treat a broad variety of bacterial infections [27]. In comparison to erythromycin, strictinin was found to possess relatively weak inhibitory activities against *Propionibacterium acnes* and *Staphylococcus epidermidis*, with minimum inhibitory concentrations of 250 mM and 2000 mM, respectively [28]. The inhibitory activities of strictinin against these two bacteria were approximately 1000 times weaker than erythromycin and the nine other antibiotics examined in the study. According to the structural comparison, the extra portion with a nitrogen atom found in erythromycin and not in strictinin might play a key role in the inhibition of bacterial infections. Nevertheless, there were additive, or synergistic effects of strictinin on the combined antibacterial activity with each of the 10 antibiotics examined. It seems that strictinin is suitable to be developed as a mild natural antibiotic and may also be safely used in combination with approved antibiotics to enhance the antibacterial effects. Accordingly, water extracts from Pu’er tea were demonstrated to possess antibacterial activities by showing inhibitory potency on the growth of Gram-positive *Staphylococcus aureus* and *Bacillus subtilis*, although the active component was not detected in the study [29]. Moreover, strictinin isomers extracted from the root of *Rosa roxburghii* Tratt (Ci Li Gen) were shown to inhibit *Escherichia coli*, and the antimicrobial mechanism was shown to be related to oxidative stress and protein synthesis disorder [30].

### 2.3. Anti-Obesity Effect 

**Figure 5:**
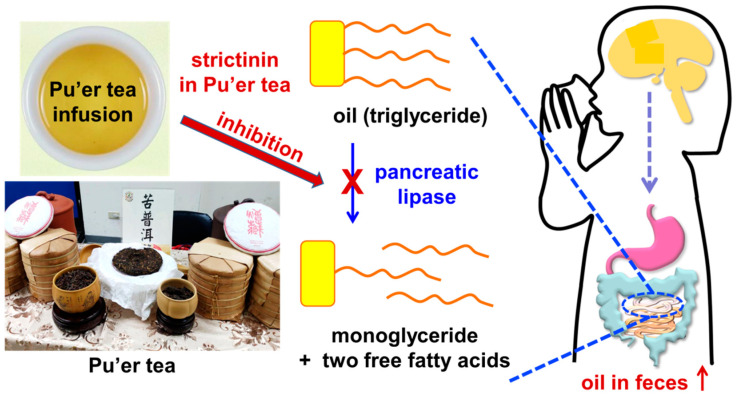
Inhibition of pancreatic lipase by strictinin in Pu’er tea. The anti-obesity effect of Pu’er tea is assumed to result from the inhibition of pancreatic lipase in the intestine. Triglyceride molecules (oil) from diets are supposed to be digested into monoglycerides and fatty acids by pancreatic lipase prior to absorption in the small intestine. Without digestion, the triglyceride molecules cannot be absorbed into the blood circulation system; thus, are expelled by defecation.

The concept to evaluate the anti-obesity effects of strictinin was initially developed from the empirical observation of tea consumers in Taiwan, whereby Pu’er tea apparently provided much better anti-obesity effects than other popular teas, such as black tea, oolong tea, and green tea prepared from different varieties of small-leaf tea species [31,32]. It was reasonable to surmise that strictinin might play a key role in anti-obesity effects since it was found as a unique major constituent in Pu’er tea, instead of a minor constituent in other teas [10]. In the first attempt, strictinin was demonstrated to exhibit an inhibitory potency on pancreatic lipase activity in a dose-dependent manner in vitro [33]. The IC_50_ value of strictinin for the inhibitory potency on pancreatic lipase was found to be 90 μg/mL. Therefore, the empirical anti-obesity effects of Pu’er tea were proposed to be a consequence of blocking fat absorption from diets by inhibiting pancreatic lipase activity in the small intestine (Figure 5). However, there is currently no scientific evidence to clarify that the inhibition of pancreatic lipase activity by strictinin resulted from its specific binding to the enzymatic active site of pancreatic lipase, or by non-specifically covering the surface of the pancreatic lipase and/or triglyceride emulsions encapsulated by bile acid in the intestine.

Following the above in vitro evidence, an animal study was employed to examine the anti-obesity effect of strictinin in vivo compared to orlistat, a commercial anti-obesity drug [34]. Similar to the fat-lowering effect of orlistat, oral administration of strictinin significantly decreased the blood triglyceride levels in mice, dose-dependently, in the olive oil tolerance test [33]. Comparably, the effectiveness of 100 mg/kg strictinin was found to be equivalent to 10 mg/kg orlistat, in terms of reducing fat absorption in mice. As expected, the oil content in the fecal excretion of mice was found to be elevated as the diet fat absorption was reduced by supplementation of strictinin. It was proposed that strictinin is an adequate candidate to be developed as a natural substitute for orlistat, which is not recommended for daily administration due to its gastrointestinal side effects and severe hepatic adverse effects [35,36].

In another animal study, the increased bodyweight of mice undertaking a high-fat diet was significantly reduced by strictinin supplementation over a long-term observation of eight weeks [33]. In this study, epididymal fat, but not liver or muscle, was found to be significantly expanded with the sizes of the adipocytes in the epididymis substantially enlarged in mice fed with a high-fat diet. The accumulation of epididymal fat as well as the enlargement of adipocytes in mice fed with the high-fat diet was substantially reduced when strictinin was supplemented daily. Furthermore, the levels of blood triglyceride, cholesterol, and glucose were apparently raised in mice fed with the high-fat diet, and these raised levels were significantly attenuated when strictinin was supplemented. Taken together, strictinin is assumed to be the key ingredient responsible for the empirical anti-obesity effects of Pu’er tea [37], presumably via the prevention of fat absorption from diets by inhibiting pancreatic lipase activity.

### 2.4. Laxative Activity

The inspiration for the identification of strictinin laxative activity came from the documentation of Pu’er tea as an effective laxative in some ancient books of traditional Chinese herbal medicine, e.g., the Compendium of Materia Medica (Bencao Gangmu). In this book, the consumption of Pu’er tea was described to possess many beneficial functions, such as removing fat, enhancing food digestion, elevating laxatives, detoxifying, relieving heat, eliminating mucus, relieving coughs, prolonging life, etc. In contrast to the strong laxative effect of Pu’er tea, unperceived or mild laxative effects were empirically experienced by the consumption of other teas, including black tea, oolong tea, and green tea. Being a unique constituent abundantly found in Pu’er tea, strictinin was reasonably speculated to be the key ingredient responsible for the laxative effect of Pu’er tea. As expected, strictinin was demonstrated to possess laxative activity in an animal study [28]. According to the study, the laxative activity of strictinin in the rats putatively resulted from the acceleration of the small intestinal transit, instead of the enhancement of gastric emptying, increase of food intake, or induction of diarrhea. Indeed, the results were in accordance with another study, described earlier, showing that strictinin was able to block fat absorption in the intestine by inhibiting pancreatic lipase activity, as the undigested triglyceride molecules might also lead to the laxative effect [33]. Evidently, strictinin is the key ingredient responsible for the laxative effect of Pu’er tea.

### 2.5. Anticaries Effect

**Figure 6:**
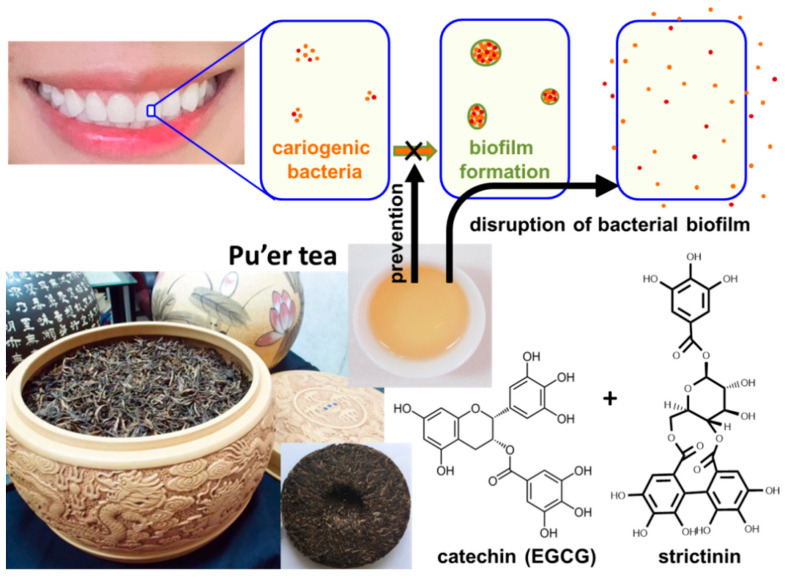
A diagram illustrating the anticaries mechanism of Pu’er tea. Pu’er tea infusion as well as its two major constituents: strictinin and (−)-epigallocatechin gallate (EGCG), prevented and disrupted biofilm formation by cariogenic bacteria.

Dental caries is a common oral disease caused by bacteria in the human oral cavity. Cariogenic bacteria, such as *Streptococcus mutans* and *Streptococcus sobrinus*, were found to be the main pathogens that play an important role in human dental caries [38]. These bacteria attach and accumulate on the tooth surface to form a sticky biofilm termed dental plaque, prior to eroding the tooth by hydrolyzing sugars into acids [39]. Around one thousand years ago, Su Dongpo, a famous Chinese writer within the Song Dynasty, documented that he constantly rinsed his mouth with heavy tea to protect his teeth. Therefore, it is common practice for some Chinese people to use tea infusion as an edible mouth rinse in their daily life. All the popular teas were empirically found to be useful for an anticaries effect, while Pu’er tea was found to be the most effective mouth rinse among the commercially available teas in Taiwan. Furthermore, strictinin was suspected to be the key constituent responsible for the superior anticaries effect of Pu’er tea. According to this suspicion, the inhibitory effects of strictinin on *S. mutans* and *S. sobrinus* were examined, with the results showing a relatively weak inhibition of bacterial growth by strictinin and (−)-epigallocatechin gallate (EGCG), a major catechin found in most tea varieties, including Pu’er tea [40]. However, biofilm formation by these two cariogenic bacteria was strongly prevented by strictinin or EGCG, while strictinin displayed a higher potency than EGCG, in preventing biofilm formation. It seems that the main mechanism of Pu’er tea in the prevention of tooth decay was in disrupting the biofilm formation of cariogenic bacteria rather than sterilizing, while this role was principally performed by strictinin and catechins (Figure 6). The data also explained why all tea varieties containing abundant catechins are effective as a mouthwash, while Pu’er tea, which is rich in both strictinin and catechins, was superior to other teas, as experienced in daily life. 

### 2.6. Anti-Allergic Activity

Interleukin 4 is an effective modulator activating the immunoglobulin class switching from IgM to IgE in B cells, and IgE is involved in the pathogenesis of allergic diseases [41]. The anti-allergic activity of strictinin isolated from fresh tea leaves was demonstrated to inhibit interleukin 4-induced STAT6 activation and antigen-specific IgE production [42]. In contrast, no detectable anti-allergic activity was observed for catechins, the abundant polyphenols in tea, under the same assay condition. Strictinin was, firstly, shown to inhibit STAT6 signaling dose-dependently in peripheral blood mononuclear cells obtained from both healthy and atopic donors. No cytotoxicity was observed for strictinin at any of the concentrations examined. In an animal study, ovalbumin-induced IgE production was reduced by strictinin supplementation, whereas the productions of IgG and IgM were not affected in mice. The data indicated that the production of antigen-specific IgE antibodies, activated by interleukin 4 in vivo, was selectively downregulated by strictinin. Furthermore, the IL-4 receptor α, in non-lipid rafts, was demonstrated to be the target of strictinin to inhibit STAT6 activation [43]. It was suggested that strictinin might be a harmless drug for treating allergic diseases.

### 2.7. Antipsoriatic Effect

Psoriasis is a skin disease that causes a rash with scaly patches frequently induced by hyperproliferation of abnormal keratinocytes in the epidermis [44]. It is also a systemic inflammatory disease [45]. In spite of being a noncontagious chronic disease with no direct threat to life, psoriasis is a risk factor in the development of other diseases, such as psoriatic arthritis and postinflammatory hypopigmentation as well as for the induction of some mental problems, such as low self-esteem and depression owing to the patients suffering from psychological pressures and social stigmas, in addition to the physical discomfort [46]. Since no medicine is currently available to extirpate psoriasis completely, steroid creams and immunosuppressive drugs are clinically applied to relieve uncomfortable symptoms [47]. It seems to be a safe and workable approach to develop novel oral medicines and topical agents by screening natural antipsoriasis compounds from edible sources, such as vegetables, fruits, beverages, and herbal medicines.

To evaluate the antipsoriatic activities of strictinin and two other major active constituents (theacrine and EGCG) in Pu’er Kucha tea, an animal model with psoriasis-like dermatitis was induced on the shaved dorsal skin of mice by topical application of imiquimod [48]. The antipsoriatic effects of Pu’er Kucha tea were observed and showed a significant reduction in the dorsal skin lesions, alleviation of splenomegaly, and attenuation of inflammatory activation. Accordingly, strictinin, theacrine, and EGCG were individually demonstrated to possess antipsoriatic effects, as observed by the significant reduction in dorsal skin lesions. Nevertheless, none of the three constituents individually exhibited antipsoriatic activity equivalent to Pu’er Kucha tea. It seemed that the antipsoriatic effects of Pu’er Kucha tea were additively contributed by strictinin, theacrine, and EGCG; splenomegaly was mainly alleviated by theacrine, while inflammatory activation was primarily attenuated by strictinin. It was also proposed that the topical application of Pu’er Kucha tea extract might be therapeutic for psoriasis treatment, although the functional compounds in the topical application and oral supplementation might be different after the metabolic modification and conversion in the human body. In this aspect, oral supplementation of Pu’er Kucha tea and the topical application of the powder or cream containing Pu’er Kucha tea extract might be simultaneously applied to psoriasis patients for an additive effect. Recently, anti-inflammatory effects of strictinin and casuarictin, two ellagitannins extracted from *Rosa roxburghii*, were demonstrated by suppressing poly(I:C)-induced IL-8 production in human keratinocytes [49].

### 2.8. Antihyperuricemia Effect

Hyperuricemia is regarded as a metabolic disease with high blood uric acid that is frequently derived from the dietary intake of high-purine food [50]. It is also noted as a major risk factor for gout and metabolic syndromes. Antihyperuricemia drugs, such as allopurinol, are clinically effective at lowering blood uric acid levels by inhibiting liver xanthine oxidase, the enzyme that generates uric acid, via oxidation of xanthine [51]. However, the side effects of allopurinol, including allergies, liver necrosis, and a decline in renal function usually upset patients during the treatment [52]. On this basis, antihyperuricemia effects by strictinin were evaluated in a cellular model by treating AML12 mouse hepatocytes with xanthine as well as in an animal model by treating mice with potassium oxonate [53]. The results showed that xanthine oxidase activity, uric acid production, and inflammation in xanthine-treated hepatocytes were significantly decreased by strictinin supplementation. For the first time, the antihyperuricemia activity of strictinin was demonstrated as resulting from anti-inflammation, via the inactivation of the NOD-like receptor family pyrin domain containing 3 (NLRP3) pathway. Correspondingly, the antihyperuricemia activities, including uric acid lowering effect and renal protection, were observed by strictinin supplementation in the potassium oxonate-treated mice. Moreover, beneficial adjustment of gut microbiota was also observed by strictinin supplementation in the animal model, indicating that strictinin might be an active ingredient to improve the gut microbiota population. It was also possible that the antihyperuricemia activities of strictinin might be partially attributed to its alteration of the gut microbiota. Taken together, strictinin is a functional ingredient for the reduction of uric acid production, inflammation, and renal damage as well as a healthy component to recuperate gut microbiota populations. Certainly, more examinations, such as the toxicity of strictinin in high dosages, should be evaluated prior to its commercialized use.

### 2.9. Antidiabetic Activity

Purple tea, a natural mutant of *Camellia sinensis*, is rich in anthocyanins and ellagitannins and has been consumed as a traditional medicine for diabetes in China, India, and African countries [54,55,56]. The antidiabetic activity of purple tea was attributed to its three major ellagitannins: strictinin, corilagin, and tellimagrandin I [57]. Molecular modeling and docking showed that these three ellagitannins as well as their metabolites, urolithin A and urolithin B, were able to bind to α-glucosidase and α-amylase, and their inhibitory potency was also confirmed by in vitro enzyme assays. Furthermore, both urolithin A and urolithin B were demonstrated to increase glucose uptake in adipocytes, muscle cells, and hepatocytes as well as reducing lipid accumulation in adipocytes and hepatocytes. It is indispensable that the proposed antidiabetic activity of strictinin, corilagin, and tellimagrandin I should be further confirmed in follow-up animal models. Comparably, antidiabetic activity was also reported for Pu’er tea, although the active ingredient was not identified [58]. 

### 2.10. Anticancer Effect

Triple-negative breast cancer (TNBC) is the most aggressive subtype of breast cancer due to its lack of hormone receptors for estrogen, progesterone, and human epidermal growth factor [59]. Therapeutics for TNBC are challenging owing to its low response to hormone treatment [60]. The non-availability of effective treatment for TNBC encouraged the attempt to screen natural compounds with anticancer activity for selective inhibition of TNBC development without severe adverse effects on non-cancer cells. On the basis of this approach, strictinin isolated from *Myrothamnus flabellifolius*, an anticancer medicinal plant found in South Africa was demonstrated to possess antiTNBC effects with minimal inhibition on the non-malignant MCF-10A breast epithelial line [61]. Further investigation into the molecular mechanism indicated that strictinin repressed TNBC survival by strongly inhibiting the receptor tyrosine kinase orphan-like1 (ROR1) receptor via the modulation of PI3K/AKT/GSK3β activity [62]. Repression of TNBC cell migration and invasion was also observed in a beta-catenin-independent manner following strictinin supplementation. Being a ROR1-inhibitor, strictinin was proposed as a potential anticancer agent. Of course, the proposed anticancer activity of strictinin needs to be further verified in animal models prior to clinical trials.

### 2.11. Summary of Functional Activities of Strictinin

**Figure 7:**
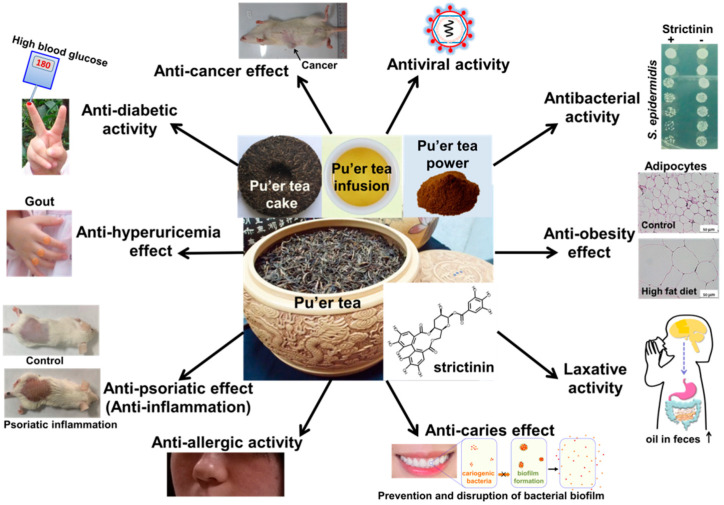
A diagram summarizing the 10 functional activities documented for strictinin.

More and more functional activities were demonstrated for strictinin recently. These functional activities were mostly in accordance with the therapeutic effects empirically perceived for Pu’er tea. The 10 functional activities described in this review are summarized in the following diagram (Figure 7). 

## 3. Application of Striction

### 3.1. Powder of Pu’er Tea Infusion

Since various activities have been demonstrated for strictinin over the past decade, highly purified strictinin is assumed to be a potential natural drug. Moreover, Pu’er tea, which is rich in strictinin, is reasonably considered to be a new raw material that can be supplemented to many health-improving products, according to its functional activities. To improve quality control, infusion of Pu’er tea is recommended to be dried in powder form prior to being used. During the concentration and powdering processes, Pu’er tea infusion should be kept at low temperatures (lower than room temperature) since strictinin is thermally unstable [10]. The powder of Pu’er tea infusion can be used directly as an instant tea product or supplemented with diverse functional products in the form of food, cosmetics, and beverages.

### 3.2. Bitter Citrus Tzen Tea 

In light of the threat of the COVID-19 outbreak and the constant overspread of influenza, my research team attempted to screen various therapeutic teas empirically consumed in relation to expelling the common cold. Three kinds of tea, bitter Pu’er Kucha tea, Hakka sour citrus tea, and Tzen tea were identified and combined to develop a new type of tea product named Bitter Citrus Tzen Tea [63]. As described in the previous section, bitter Pu’er Kucha tea was consumed as an herbal tea by Aboriginal people in Yunnan, China when experiencing a cold. Moreover, caffeine and catechins are abundantly found in most tea varieties, while strictinin and theacrine were found as extra major constituents in bitter Pu’er Kucha tea and demonstrated antiviral activity [16]. Since the antiviral activity of strictinin was higher than theacrine, the content of strictinin was taken as a key index in the quality control of the Bitter Citrus Tzen Tea.

Hakka sour citrus tea was originally prepared by the Hakka people when they lived in the mountain areas of Hsinchu, Taiwan, and almost all families regularly kept the tea at home to expel the cold since it was not convenient for them to visit a doctor at that time. The peel of a hybrid citrus species, *Citrus aurantium* L. cv. Hutou Gan was used as the key material in Hakka sour citrus tea, and two flavonoid glycosides, naringin, and hesperidin, known to possess anti-inflammatory, anti-influenza, antitussive, and expectorant activities were identified as the major constituents [64,65,66,67,68]. 

According to folklore as well as descriptions in ancient literature, old tea is regarded as a herbal medicine to cure several diseases, including the common cold. Unfortunately, no standard samples of old tea with medicinal functions are available nowadays, and the precise protocol for the preparation of old tea by baking and aging has never been documented. To solve the mystery of this ancient process technology and to assist in the production of a functional drink in a scientific manner, Tzen tea was developed as a new type of tea refined from fresh oolong tea after baking and aging periodically [69]. In comparison to the original oolong tea, Tzen tea was noted with a significantly reduced caffeine content (by approximately 80%), the astringent sensation substantially removed, and the umami taste (savory taste) apparently enhanced, while its active ingredient, teaghrelin was mostly retained (>80%). Teaghrelins are unique acylated flavonoid tetraglycosides, currently found in Chin-shin and Shy-jih-chuen oolong tea, and have been demonstrated to increase appetite, accelerate gastric emptying, enhance growth hormone secretion, prevent neurodegenerative disease, and to inhibit muscle atrophy, presumably by binding to the ghrelin receptor [70,71,72,73,74].

### 3.3. Edible Oral Care Products

Some chemicals, such as chlorhexidine, fluoride, and antibiotics have been clinically used to treat and prevent caries, although side effects were observed in patients treated with these anticaries chemicals [75,76,77]. Recently, allergy cases to chlorhexidine have been increasingly reported [78]. Although topical fluoride application in the tooth cavity seems to be an effective method for caries prevention, systemic fluoride uptake might cause adverse effects, including neurotoxicity [79]. Long-term utilization of antibiotics tends to change microbial flora in the human body, which sometimes leads to antibiotic resistance, one of the current serious global health threats [80]. Therefore, natural compounds with safe anticaries effects seem to be an alternative solution in the prevention of human dental caries. According to the inhibitory potency of strictinin on cariogenic bacteria, purified strictinin, as well as Pu’er tea extract, may be beneficially supplemented to anticaries products, which are used to gargle for the prevention of tooth decay. As the strictinin content in Pu’er tea varies significantly in different sources, those Pu’er teas that are rich in strictinin (>2% by dry weight) appear to be a new type of functional raw material, which can be supplemented to oral care products, such as toothpaste and edible mouthwash.

## 4. Conclusions and Future Perspectives

Over the past decade, strictinin has been demonstrated to be a valuable natural compound with many functional activities. The abundant content of strictinin is regarded as a unique characteristic of Pu’er tea plants in comparison to other tea varieties and plant species. The therapeutic effects empirically perceived for Pu’er tea seem to be mostly attributed to its high strictinin content. Several functional activities of strictinin, such as its antiviral, antibacterial, anti-obesity, and anticaries effects might be explained by its surfactant chemical competence prior to its absorption into the blood circulation system. In contrast, some other activities by strictinin, such as its anti-allergic, antipsoriatic, antihyperuricemia, antidiabetic, and anticancer effects were putatively activated after its uptake into the human body. The molecular mechanisms responsible for the functional activities need to be elucidated in detail. The bioavailability of strictinin and the kinetics of its metabolites—urolithins—are suggested to be detected in clinical trials. Moreover, visualization techniques, such as chemical or physical imaging and immunological staining should be developed to track strictinin in vivo and in vitro. To expedite the commercial availability of strictinin-based products, manufacturing for the massive production of edible powder from Pu’er tea should be optimized to stably supply this functional constituent at a reasonable cost. Judging from current progress, it is anticipated that more functional activities and practical applications of strictinin will be scientifically reported in the not-too-distant future.

## Data Availability

Not applicable.
